# Oral manifestations of sickle cell disease and its effects on dental and periodontal health: A systematic review

**DOI:** 10.34172/japid.025.3467

**Published:** 2025-04-13

**Authors:** Mouhamadou Lamine Guirassy, Folly Emmanuel Baglo, Ahmad Moustapha Diallo, Ndeye Lira Mbow, Diabel Thiam, Adam Seck-Diallo, Henri Michel Benoist

**Affiliations:** Service of Periodontology, Institute of Odontology and Stomatology (IOS), Faculty of Medicine, Pharmacy and Odontostomatology, Cheikh Anta Diop University of Dakar, Senegal

**Keywords:** Oral health, Periodontal diseases, Sickle cell anemia, Sickle cell disease

## Abstract

**Background.:**

Sickle cell disease is a genetic disorder that manifests itself in several organs. There is little consensus in the literature on oral manifestations, particularly dental and periodontal. This study aimed to identify the oral manifestations of sickle cell disease, focusing on dental and periodontal manifestations.

**Methods.:**

An electronic search was performed in PubMed, Embase, and African Index Medicus. Quality and risk of bias were assessed using the Newcastle-Ottawa Scale, the modified Newcastle-Ottawa Scale, and the 2013 Guideline CARE. This systematic review covered research published between 2000 and 2022.

**Results.:**

A total of 962 articles were identified, 26 of which were selected, including 13 case–control studies, 4 cohort studies, 7 cross-sectional studies, and 2 case reports. The risk of bias was high for 3.84% of the studies, medium for 38.46%, and low for 57.60%. Oral manifestations were reported in 24 studies, with a predominance of periodontal ones in 10 studies. An association between sickle cell disease and dental caries, pulpal necrosis, and delayed tooth eruption has been reported.

**Conclusion.:**

Several oral manifestations, particularly periodontal, of sickle cell disease have been reported. However, current data do not provide evidence of a possible association between sickle cell disease and oral symptoms, particularly periodontal manifestations.

## Introduction

 Sickle cell anemia is one of the world’s most common monogenic disorders.^1^ It is caused by a mutation in the beta-globin gene’s sixth codon, which leads to the synthesis of an abnormal hemoglobin called hemoglobin S (HbS).^2^ The mutation can be heterozygous (Hb AS), homozygous (Hb SS), or combined with other hemoglobin defects, such as the -globin gene (Hb SThal), to cause sickle cell trait (SCT) and beta-thalassemia (T). Mutant hemoglobin results in sickle-shaped erythrocytes in hypoxic conditions, causing blood vessel obstruction and tissue necrosis.^1,3^

 According to the World Health Organization (WHO), around 5% of the world’s population carries a gene causing a hemoglobin anomaly, and around 50 million people are affected, with severe forms predominating in equatorial Africa (15%–30%).^3-5^ Due to the global migration of populations, sickle cell disease has become a public health issue.

 The disease is characterized by hemolytic anemia, vaso-occlusive phenomena, and susceptibility to infection, with frequent acute or chronic complications.^1^ These include pain, cerebral complications, heart failure, severe infections such as sepsis, osteomyelitis, and vaso-occlusive crises.^1-6^

 Some oral manifestations of sickle cell disease have been described, including increased levels of biofilm, aseptic pulpal necrosis, decreased salivary flow, paleness of the oral mucosa, delayed tooth eruption, enamel hypoplasia, and oral neuropathies.^7-11^ Dental caries and periodontal disease can be a source of infection in sickle cell crises.^8,11^ Gingival bleeding and biofilm have been noted in sickle cell patients with decreased salivary flow.^12^ de Carvalho et al^13^ reported that SCT is associated with gingivitis and periodontitis. Mahmoud et al^14^ observed a significantly higher prevalence of inflammatory periodontium in children with sickle cell disease compared with controls without sickle cell disease. However, other studies have reported no oral manifestations in patients with sickle cell disease.^10,15^ There is no consensus on the oral manifestations of sickle cell disease and its effects on dental and periodontal health. A synthesis of current data on the dental and periodontal manifestations of sickle cell disease is needed to establish a prevention and management policy. The present study was undertaken to identify the oral manifestations of sickle cell disease and its effects on dental and periodontal health.

## Methods

 This systematic review was conducted according to PRISMA statement guidelines.^16^ The research question was: “What are the dental and periodontal manifestations of sickle cell disease?” This question has been articulated as follows (P ₌ population; E ₌ exposure; O ₌ outcome):

- Population: sickle cell patients
- Exposure: oral manifestations of sickle cell disease
- Outcome: effects of oral manifestations on dental and periodontal health

###  Search strategy and identification of relevant studies

 An article search was carried out using Pubmed, Embase, and the African Index Medicus. “anemia, sickle cell,” “sickle cell disease,” “hemoglobin SC disease,” “oral diseases,” “periodontal diseases,” “dental health,” “dental caries,” “dental pulp disease,” and “dental abnormality” were the MeSH designations and keywords used. The Boolean operators “AND” and “OR” were used to combine these words. Over one month, a manual search was also conducted in the journals of *Periodontology and Oral Implantology*, the *International Journal of the African College of Odontology and Maxillofacial Surgery*, and the *Journal of Tropical Odontology*.

###  Inclusion and exclusion criteria

 This systematic review included research published in both English and French from 2000 to 2022. The included articles were case–control studies, cohort studies, cross-sectional studies, and case reports. Expert letters, opinions, animal research papers, systematic reviews, and experimental studies were excluded.

###  Study selection

 After implementing the search strategy, the results were fed into the Rayyan program (Rayyan.ai). After removing duplicates, two independent reviewers examined all the collected studies for inclusion. During this phase, articles with irrelevant titles or abstracts were excluded, and the full texts of the selected articles were examined. Two separate reviewers subsequently assessed the full texts to determine their final inclusion. Discrepancies were handled by a third reviewer after anonymity was removed (Rayyan).

###  Quality assessment of studies 

 Two independent reviewers assessed the quality of the included studies. The Newcastle-Ottawa Scale (NOS) was used to assess the quality of cohort and case–control studies. This scale takes into account selection, comparison, and exposure. These three key areas were used to provide a maximum of 9 points to the quality of cohort and case–control studies. Cross-sectional studies were graded using the modified NOS, which assigns a maximum of 10 stars to three areas (selection, comparability, and results). To evaluate the quality of case reports, the 2013 CARE Guideline was used. The introduction, discussion, and conclusion are all rated on this scale. These three key areas were used to assign a maximum of 9 points to case reports and case series.

###  Data extraction

 FEB and AMD extracted the data. The parameters collected from each study were the identity of the first author, year of publication, country of study, type of study, sample size, mean age, means of diagnosis of sickle cell disease, oral manifestations studied, and the main results.

## Results

###  Study selection

**Figure 1:**
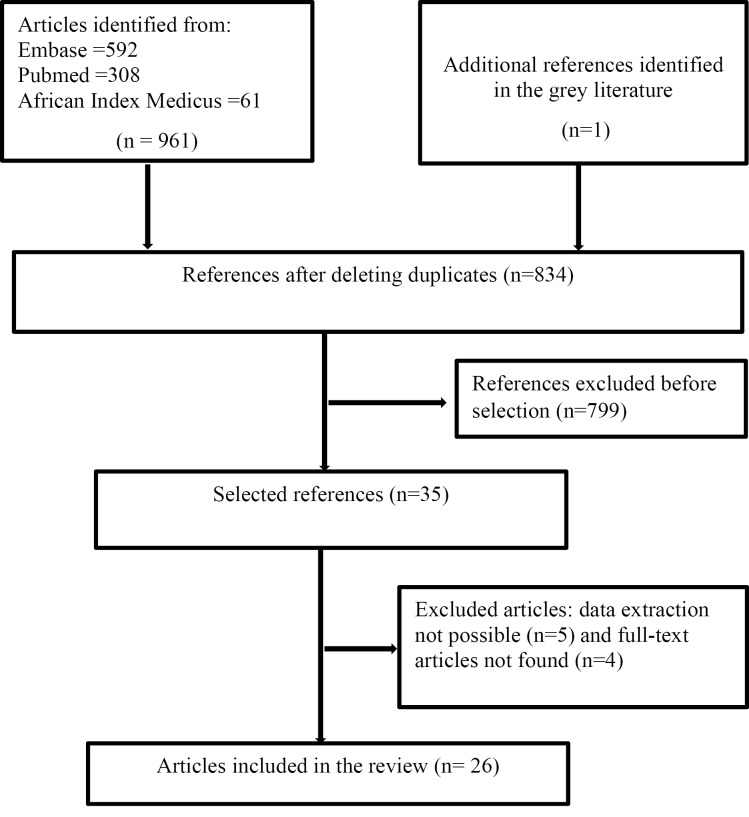
PRISMA chart of the manuscript selection process

Figure 1 depicts the flowchart of the study selection process. PubMed, Embase, African Index Medicus, and grey literature yielded 308, 592, 61, and 1 article, respectively. We reviewed 35 full-text articles; however, 4 full-text articles were not retrieved, and data extraction was not possible for 5 articles. In the end, we included 26 studies in the systematic review.

###  Characteristics of the included studies

Table 1 summarizes the key features of the included research. The articles chosen were published between 2001 and 2022 and included 13 case–control studies, 4 cohort studies, 7 cross-sectional studies, and 2 case reports. Most studies used electrophoresis to diagnose sickle cell disease, whereas others used medical history or medical records, high-performance liquid chromatography, genetics, and molecular biology. Cold heat test, gingival index, sulcular bleeding index, plaque index (PI), periodontal probing depth (PPD), clinical attachment loss (CAL), bleeding on probing (BOP), community periodontal index (CPI), and decayed, missing or filled teeth index (DMFT) were used to define oral manifestations. Most studies included in the review reported oral symptoms of sickle cell disease. Only two studies showed no link between sickle cell disease and the outlined oral symptoms. Several oral signs of sickle cell disease were recorded, with periodontal manifestations predominating in 10 of the 26 included studies.

###  Quality assessment 

Tables 2, 3, 4, and 5 summarize the quality assessment of the included studies. Cohort studies had scores ranging from 7 to 8 (good quality) out of 9 (Table 2). Of 13 case–control studies, only one had a score of 4/9; all the others had scores ≥ 6/9, ranging from 6 (average quality) to 9 (good quality, Table 3). As for the cross-sectional studies, their scores ranged from 5 to 9 out of 10, i.e., from moderate risk of bias (5) to low risk of bias (9, Table 4). The evaluation of case reports resulted in scores ≥ 5/9 (Table 5).

**Table 1:** Characteristics of the studies included

**Authors, year (country)**	**Type of study**	**Sample size**	**Average age** **(years)**	**SCD Diagnostics**	**Events studied**	**Main results**
Scipio et al, 2001^17^ (Trinidad and Tobago)	Case report	1 SCD	14	Medical history	Facial swelling, Gingival hypertrophy	Swelling due to extravasated blood and gingival hypertrophy suggests numerous attempts at fibrous repair.
Demirbaş Kaya et al, 2004^18^ (Turkey)	Case–control	72 36 HbSS 36 without SCD	From 16 to 40 years old	Electrophoresis	Orofacial and dental pain, Pulpal necrosis, Bone quality	67% of SCA patients with vital teeth had an OFDP with no obvious cause. A statistically significant difference between SCA and control groups (*P* < 0.05) in terms of pulpal sensitivity.
Oredugba, 2005^19^ (Nigeria)	Case report	1 HbSC	15	Medical history	Hypodontia	Genetic link between HbSC and hypodontia, although this has not previously been reported in the literature.
Benoist et al, 2006^20^ (Senegal)	Cross-sectional	50 HbSS	9.4 ± 3.8	Medical records	Gum inflammation, Oral hygiene	66% of SCD have severe inflammation, which is more frequent in the 3 to 12 age group, and 68% have bleeding, which is moderate to severe in 18%, with a greater frequency in the 13 to 16 age group.
Laurence et al, 2006^10^ (United States)	Case–control	205 102 SCD 103 without SCD	From 18 to 70 years old	Electrophoresis Medical records	Dental decay	SCD patients had more decayed tooth surfaces than subjects without SCD. The difference was statistically significant (*P* < 0.05) after adjustment for age and sex.
Licciardello et al, 2007^21^ (Italy)	Case–control	72 36 HbSS 36 without SCD	36 HbSS (14 βs βs 28 ± 5.9, 13 βs β0th 27.5±8 9βs β+th 32.8±9.9) 28.9 ± 8	Hematological Molecular Genetic	Craniofacial anomalies, Orthodontic anomalies	More pronounced posterior rotation of the mandible in SCD subjects. All patients had significantly more proclination of the maxillary incisors than in the control group.
Mendes et al, 2011^22^ (Brazil)	Case–control	330 165 HbSS 165 without SCD	16.4 ± 11.5 18.2 ± 14.4	Medical records, Medical history	Pallor of the oral mucosa, Delayed tooth eruption, Mandibular osteomyelitis, Anterior mental nerve disease, Orofacial pain	SCD patients had a significantly higher prevalence of anterior mental nerve disease (*P* = 0.000) than patients without SCD
Passos et al, 2012^23^ (Brazil)	Case–control	190 99 SCD (51 HbSS et 48 HbSC) 91 without SCD	32.66 ± 11.62	Electrophoresis	Dental decay, Periodontal disease	The mean number of decayed dental was significantly higher in subjects with HbSS than in the control group (*P* = 0.01). There was no statistically significant difference in mean CPI between the two groups.
Costa et al., 2013^24^ (Brazil)	Cohort	350 124 SCD 226 without SCD	26 (median)	Electrophoresis	Pulpal necrosis	The occurrence of PN in clinically intact permanent teeth was 8.33 times higher in the exposed group than in the unexposed group (*P* < 0.001).
Mahmoud et al, 2013^14^ (Sudan)	Case–control	113 59 SCD 54 sans SCD	14.03 ± 1.4 13.91 ± 1.38	Electrophoresis	Periodontal disease	A statistically significant association between GI and SCD (P = 0.002). The percentage of teeth with PD = 4 mm was 2.5% in SCD patients and 0.6% in controls. SCD patients had a higher percentage of teeth with CAL = 3 mm (0.7% vs. 0.3%). A statistically significant association between mild, moderate, or severe GI and SCD severity (*P* = 0.028).
Singh et al, 2013^15^ (India)	Case–control	750 500 (250 BT; 250 HbSS/C) 250 without SCD	From 3 to 15 years	Medical history	Dental decay, Periodontal disease	The prevalence of DD and PD was significantly higher in BT patients, followed by SCD patients than in the control group.
Veiga et al, 2013^25^ (Brazil)	Case–control	25 10 SCD (HbSS) 15 without SCD	10.58	Electrophoresis	Periodontal disease	Higher levels of IFN-γ, TNF-α, IL-4, -5, -8, -10, and -13 only in the SCA group (*P* < 0.005). A positive correlation between BOP and IL-10 was observed.
Costa et al, 2015^26^ (Brazil)	Cohort	279 93 SCD 186 without SCD	26.0 ± 0.0 26.0 ± 9.0	Electrophoresis, Medical records	Dental malocclusion	SCD was associated with moderate (RR = 1.36) and very severe (RR = 8.0) malocclusion. SCD was correlated with anterior crossbite (RR = 1.94) and overbite (RR = 1.94).
Al-Alawi et al, 2015^27^ (Saudi Arabia)	Case–control	66 33 SCD 33 without SCD	24.52 ± 4.611 24.58 ± 6.124	Medical records	Dental decay, Periodontal diseases	Significantly decayed teeth in SCD than in the control group (*P* = 0.036).
de Carvalho et al, 2016^13^ (Brazil)	Cohort	369 123 HbSS 123 HbAS 123 without SCD	17 ± 13 33 ± 8 36 ± 19	Electrophoresis	Periodontal diseases	No periodontal parameters were associated with SCA. SCT was associated with gingivitis (P = 0.041) and periodontitis (*P* = 0.002).
Ferreira et al, 2016^28^ (Brazil)	Cross-sectional	108 SCD (HbSS)	From 5 to 59 years old	Medical records	Endodontic diseases	Correlation between SCA and ED with a significant difference between the number of eosinophils and atypical lymphocytes compared with ED.
Lisboa et al, 2016^29^ (Brazil)	Case–control	40 20 SCD 20 without SCD	From 18 to 45 years old	Medical history	Post-bleaching tooth sensitivity	72.5% of volunteers had provoked and/or spontaneous sensitivity. The factor most often mentioned as triggering sensitivity was "talking" (50%).
de Carvalho et al, 2017^30^ (Brazil)	Case–control	246 123 SCD 123 without SCD	From 12 to 52 years old From 12 to 64 years old	Medical records	Atresia of the pulp chamber, Hypotaurodontist, Alteration of the trabecular bone and lamina dura	Dental changes did not differ between groups (*P* > 0.05). The prevalence of hypotaurodontism was twice as high in SCA patients as in controls (*P* = 0.086).
Lopes et al, 2018^31^ (Brazil)	Cross-sectional	56 SCD	9.32	Medical records	Enamel defects, Delayed tooth eruption	The most common type of enamel defect was diffuse opacity (6.2%). Enamel defect was higher in men (36.7%, *P* > 0.05). The prevalence of enamel defect was high, increased with age, and was similar between the sexes.
Basyouni, et al, 2018^32^ (Saudi Arabia)	Cross-sectional	236 112 SCD 124 without SCD	15.6 ± 1.7 16.2 ± 1.9	Hematological, Molecular, Genetic	Orthodontic anomalies	In SCD patients, incisal crowding (72.4%), overhang (67.3%), and maxillary misalignment in the anterior segment (56%) were the most common types of malocclusions and were significantly higher than in controls (*P* < 0.05).
Brandão et al, 2018^12^ (Brazil)	Cross-sectional	124 61 SCD 63 (comparison)	12.4 ± 2.9 11.1 ± 2.9	Electrophoresis, High-performance liquid chromatography	Dental decay, Periodontal disease, Salivary flow quality	Periodontal examinations showed the presence of GB and tartar, with no significant difference between the groups (*P* = 0.984). The DMFT was 2.08 (2.71) for the SCD group and 1.05 (1.67) for the comparison group (*P* = 0.013).
Kalbassi et al, 2018^33^ (Iran)	Cross-sectional	275 120 BT 55 SCD 100 without SCD-BT	18,8 ± 1.124 19.2 ± 2.91 19.3 ± 3.211	Medical records	Dental decay, Periodontal disease, Orthodontic anomalies, Pallor of the oral mucosa	Significantly higher prevalence (*P* < 0.05) of oral manifestations in BT patients (GI = 2.18 ± 1.300, 1.64 ± 0.963; Decayed teeth = 8.31 ± 3.330, 2.33 ± 1.221; Missing teeth = 3.51 ± 2.016, 1.19 ± 0.820; DMF = 13.92 ± 7.001, 2.63 ± 1.301) in BT and non-BT patients respectively.
Souza et al, 2018^34^ (Brazil)	Cohort	369 123 SCD 123 SCT 123 without SCD-SCT	Median age 17 ± 13 33 ± 8 36 ± 19	Medical records	Pulpal calcification, Changes in the RSP, Alterations in the trabecular meshwork, Alterations in the lamina dura	In SCA patients, there is a higher number of teeth with PC. In SCT patients, there is a higher number of teeth with hypercementosis.
Carvalho et al, 2020^35^ (Brazil)	Cross-sectional	686 SCD	37.8 (months)	Medical records	Dental decay	Pain crises and hospitalizations were positively associated with dental decay (crude OR = 2.11 and adjusted CR = 1.24; crude CR = 2.50 and adjusted CR = 1.46, respectively), but these associations were not statistically significant.
Menka et al, 2021^36^ (India)	Cross-sectional	75 SCD	From 8 to 16.5 years old	Medical records	Dysharmonia-dento-maxillary	The majority of SCD patients had Angle Class II malocclusion.
Tonguç et al, 2022^37^ (Turkey)	Case–control	86 43 SCD 43 without SCD	From 5 to 18 years old	Medical history	Periodontal inflammation, Salivary quality	Positive correlations between salivary IL-6 levels and serum Hs-CRP levels (r = 0.303, *P* < 0.05). Salivary levels of IL-6, TNF-α, and NO were increased 3 to 6-fold in children with a history of painful attacks compared with children who had never had a painful attack.

SCD: sickle cell disease; HbSS: hemoglobin type SS; HbSC: hemoglobin type SC; SCA: sickle cell anemia; OFDP: orofacial and dental pain; BT: beta-thalassaemia; CPI: community periodontal index; PN: pulpal necrosis; GI: gingival index; PD: pocket depth; CAL: clinical attachment loss; CD: dental decay; BOP: bleeding on probing; TNF-α: tumor necrosis factor-alpha; IFN-γ: interferon-gamma; IL: interleukin; RR: relative risk; SCD (βsβs): sickle cell disease βsβs; DMFT: decayed, missing and filled teeth index; HbAS: hemoglobin type AS; SCT: sickle cell trait; ED: endodontic diseases; GB: gingival bleeding; DMF: decayed, missing and filled; PC: pulpal calcification; Hs-CRP: highly sensitive serum C-reactive protein; RSP: root surface and periapex; OR: odds ratio; NO: nitric oxide.

**Table 2:** Quality assessment of cohort studies

**Study**	**Selection**				**Comparability**		**Exposure**			**Total**
Costa et al, 2013^24^	+	+	+	−	+	+	+	+	+	8
Costa et al, 2015^26^	+	+	+	+	−	+	+	+	−	7
de Carvalho et al, 2016^13^	+	+	+	+	−	+	+	+	−	7
Souza et al, 2018^34^	+	+	+	+	−	+	+	+	−	7

1: adequate definition of cases, 2: representativeness of cases, 3: selection of controls, 4: definition of controls, 5: comparability of cases and controls based on age, 6: comparability of cases and controls based on other factors, 7: determination of exposure, 8: same methods for evaluating cases and controls, 9: non-response rate.

**Table 3:** Quality assessment of case-control studies

**Study**	**Selection**				**Comparability**		**Exposure**			**Total**
Demirbaş Kaya et al, 2004^18^	+	+	+	+	−	+	+	+	−	7
Laurence et al, 2006^10^	+	+	+	−	+	+	+	−	−	6
Licciardello et al, 2007^21^	+	+	+	+	+	−	+	−	−	6
Mendes et al, 2011^22^	+	+	+	+	+	−	+	+	−	7
Passos et al, 2012^23^	+	+	+	+	+	+	+	−	+	8
Mahmoud et al, 2013^14^	+	−	+	+	+	−	+	−	+	6
Singh et al, 2013^15^	+	+	+	−	+	−	+	+	−	6
Veiga et al, 2013^25^	−	+	+	+	+	+	+	+	−	7
Al-Alawi et al, 2015^27^	−	+	+	−	+	+	+	+	−	6
Lisboa et al, 2016^29^	−	+	−	−	+	+	−	+	−	4
de Carvalho et al, 2017^30^	+	+	+	+	+	+	+	−	−	7
Basyouni, et al, 2018^32^	+	+	+	+	+	−	+	+	−	7
Tonguç et al, 2022^37^	+	+	+	+	+	−	+	−	−	6

**Table 4:** Quality assessment of cross-sectional studies

**Study**	**Selection**				**Comparability**	**Outcomes**		**Total**
Benoist et al, 2006^20^	−	−	*	**	*	**	*	7
Ferreira et al, 2016^28^	−	−	*	*	*	**	*	6
Lopes et al, 2018^31^	−	−	*	**	**	**	*	8
Brandão et al, 2018^12^	−	*	*	**	**	**	*	9
Kalbassi et al, 2018^33^	*	−	*	−	**	**	*	7
Carvalho et al, 2020^35^	−	*	−	**	**	**	*	8
Menka et al, 2021^36^	−	−	*	*	*	**	−	5

The modified NOS has been adapted for cross-sectional studies: selection (maximum 5 stars), comparability of patients concerning risk factors (maximum 2 stars), and evaluation of results (maximum 3 stars).

**Table 5:** Quality assessment of case reports and case series

**Study**	**Introduction**				**Discussion**		**Conclusion**			**Total**
Scipio et al, 2001^17^	+	+	+	+	+	−	+	−	−	6
Oredugba, 2005^19^	+	+	+	+	−	−	−	+	−	5

The 2013 CARE Guideline was used to assess the quality of case reports: 1: study relevance, 2: patient information, 3: clinical outcomes, 4: diagnostic approach, 5: therapeutic intervention, 6: follow-up and outcomes, 7: limitations and strengths, 8: patient perspectives, 9: patient informed consent.

## Discussion

 This systematic review only included studies written in French or English. Case reports or case series could weaken the level of evidence of the association found, as these are not ideal for testing evidence of association. The same applies to cross-sectional studies. However, they make it possible to identify factors without being able to rule on their causal role or to specify whether they predate or post-date the events. Nonetheless, most studies have attempted to control for cofactors, allowing associations to be confirmed. The indices used to define the presence of periodontal inflammation and dental caries varied from one study to another, making comparability difficult. This is also true for the multiple techniques used to diagnose sickle cell disease.

 In our review, 57.60% of the studies had a low risk of bias, 38.46% had a medium risk of bias, and 3.84% had a high risk of bias. The high risk of bias might be explained primarily by mistake in heading one of the assessment tools, which refers to the selection process and includes items such as case definition, case representativeness, control selection, and control definition. The high rate of studies with a low risk of bias could be explained by the studies’ methodological rigor.

 Ten of the 26 studies evaluated sickle cell disease’s periodontal symptoms. Six studies found a link between sickle cell disease and periodontal disease (PD), with PD identified simultaneously and under the same conditions as other oral diseases.^12,13,23,27,33,37^ Four studies focused only on periodontal symptoms and found a link to sickle cell disease.^13,14,20,25^This systematic review showed a predominance of periodontal disease in patients with sickle cell disease. However, this conclusion must be qualified because of the heterogeneity of the periodontal indices used. Indeed, periodontal disease has been defined in different ways: PI, gingival index, sulcular bleeding index, CPI, pocket depth, clinical loss of attachment, and serum cytokine levels. Some studies have found a positive association between sickle cell disease and gingival index but not between sickle cell disease and PI.^14^ On the other hand, de Carvalho et al^13^ found no association between periodontal parameters and sickle cell disease but reported that SCT was associated with gingivitis and periodontitis. Furthermore, some studies did not dissociate gingivitis and periodontitis in PD.^15,23,27,37^However, others identified a positive association between sickle cell disease and gingivitis but not with periodontitis.^12,25^ These authors attributed gingival hypertrophy to repeat episodes of hemorrhage and tissue repair (blood extravasation) on the one hand and elevated levels of cytokines (interleukin-6, TNF-α) and salivary nitric oxide in sickle cell disease patients on the other, which may promote PD.^17,37^ In addition, patients with sickle cell disease often have an increased inflammatory response that can intensify the gingival response to a minimal amount of biofilm, putting them at increased risk of developing PD.^8^ Other factors that may influence the occurrence of PD, such as social status, severity of sickle cell disease, and level of oral hygiene, need to be considered; however, this was not the case in the studies included in this systematic review.^38,39^

 Seven studies evaluated dental caries as an oral manifestation of sickle cell disease. A positive association was reported in all but one study, although the mean number of decayed teeth was significantly higher in subjects with sickle cell disease.^27^ Laurence et al.^10^ reported that sickle cell patients had more decayed tooth surfaces than healthy subjects, with a statistically significant difference (*P* < 0.005) after adjustment for age and sex. Carvalho et al^35^ showed that sickle cell pain attacks were positively associated with dental caries. In contrast, Yue et al,^40^ in their meta-analysis including 9 studies on the association between sickle cell disease and dental caries, indicated that the DMFT and decayed, missing, and filled surface (DMFS) index scores were not significantly different between sickle cell disease patients and healthy participants. They concluded that sickle cell patients did not suffer from more severe forms of dental caries than healthy people.

 Sickle cell disease is a risk factor for pulpal necrosis in clinically intact teeth, which can be explained on the one hand by vaso-occlusive crises in sickle cell disease, which lead to ischaemic necrosis of pulp tissue even in the absence of other risk factors and on the other hand by the vulnerability of the dental nerve when it passes through a narrow bone canal in sickle cell disease.^18,24,28^

 A positive association between sickle cell disease and delayed tooth eruption has been reported.^31,36^ This condition may be associated with the same factors that determine somatic growth retardation in sickle cell disease.

 In a cross-sectional study, Lopes et al^31^ showed that enamel defect was common in sickle cell patients and increased with age. The most common enamel defect was found to be diffuse enamel opacity. Pathological and/or medical disorders such as changes in oxygen saturation, antibiotics, infection, excessive fluoride exposure, and malnutrition might all lead to these symptoms.

 Oredugba^19^ documented hypodontia in an adolescent with SC sickle cell disease, suggesting a genetic link between the two diseases, though this has not been validated in the literature. Similarly, hypotaurodontism has been linked to hereditary disorders and is thought to be associated with sickle cell disease.^30^

 Licciardello et al^21^observed increased posterior rotation of the mandible and prominence of the maxillary incisors in patients with sickle cell disease, suggesting craniofacial growth anomalies associated with the disease.

 Despite the absence of a significant association, Souza et al^34^ reported a high prevalence of pulpal calcification and external root resorption in sickle cell patients. Pulpal calcification can be due to an accumulation of sickle cells, leading to thrombosis of the blood vessels that supply the afflicted area. External resorption is possibly related to the propensity for infection in sickle cell disease, promoting the inflammatory mechanisms associated with dental tissue damage.^34^

 This systematic review is marked by the heterogeneity of the means of diagnosis of sickle cell disease observed even within four studies that used two different means of diagnosis for the same population.^10,22,26,32^ However, for the same population, it is desirable to use a single diagnostic method for all participants to obtain reliable results. Seven studies diagnosed sickle cell disease based on medical history, with a high risk of bias, which could influence the reliability of the associations mentioned.^15,17,29,36,37,41,42^ Sickle cell disease was diagnosed in 1/3 of the studies included based on patients’ medical records, and the methods used were not specified in these studies. Electrophoresis was used in nine studies, demonstrating the reliability of the diagnosis of sickle cell disease and, consequently, the association evoked.

## Conclusion

 This study reported oral manifestations of sickle cell disease, with a predominance of its effects on periodontal health. Given the limitations of this study, the various reported dental and periodontal manifestations of sickle cell disease should be taken with caution. Studies using the same periodontal indices (PI, BOP, PPD, CAL, and alveolar bone loss) and the same diagnostic tools for sickle cell disease are needed to clarify the association between sickle cell disease and oral symptoms, particularly periodontal.

## Competing Interests

 The authors declare that they have no financial and non-financial competing interests concerning the publication of their work during submission.

## Consent for Publication

 Not applicable.

## Data Availability Statement

 The authors confirm that the data supporting the findings of this study are available within the article.

## Ethical Approval

 Not applicable.
